# Supplementary material for: A Scoping Review Evaluating the Current State of Gut Microbiota Research in Africa
Source: Microorganisms. 2023 Aug 20;11(8):2118. doi: 10.3390/microorganisms11082118 (PMC10458939; doi:10.3390/microorganisms11082118)
Supplement: Supplementary file 1 [file microorganisms-11-02118-s001.zip › Supplementary S2- Statistical analyses .pdf]

| Disease under study and whether it is of public health significance or not | Freq. | Percent | Cum. |
|---|---|---|---|
| Acute &/or viral gastroenteritis | 2 | 1.30 | 1.30 |
| Alzheimer's Disease | 1 | 0.65 | 1.95 |
| Anaemia and/or Malnourishment | 2 | 1.30 | 3.25 |
| Atopic dermatitis &/or food sensitiza.. | 2 | 1.30 | 4.55 |
| Autism spectrum disorder | 2 | 1.30 | 5.84 |
| Breast cancer & non-malignant breast .. | 2 | 1.30 | 7.14 |
| COVID-19 | 1 | 0.65 | 7.79 |
| Chronic Kidney Disease (CKD) | 1 | 0.65 | 8.44 |
| Colorectal cancer | 8 | 5.19 | 13.64 |
| Diabetes | 6 | 3.90 | 17.53 |
| Diarrhoeal diseases | 4 | 2.60 | 20.13 |
| Environmental enteric dysfunction | 2 | 1.30 | 21.43 |
| HIV- human immunodeficiency virus | 22 | 14.29 | 35.71 |
| Hepatitis C Virus (HCV) | 1 | 0.65 | 36.36 |
| Kwashiorkor &/or Marasmus | 1 | 0.65 | 37.01 |
| Malaria | 2 | 1.30 | 38.31 |
| Multiple sclerosis | 2 | 1.30 | 39.61 |
| No disease studied | 66 | 42.86 | 82.47 |
| Other | 11 | 7.14 | 89.61 |
| Posttraumatic Stress Disorder and Tra.. | 2 | 1.30 | 90.91 |
| Schistosomiasis | 4 | 2.60 | 93.51 |
| Severe Acute Malnutrition | 5 | 3.25 | 96.75 |
| Sickle cell disease/ anaemia | 3 | 1.95 | 98.70 |
| Tuberculosis | 2 | 1.30 | 100.00 |
| Total | 154 | 100.00 | |

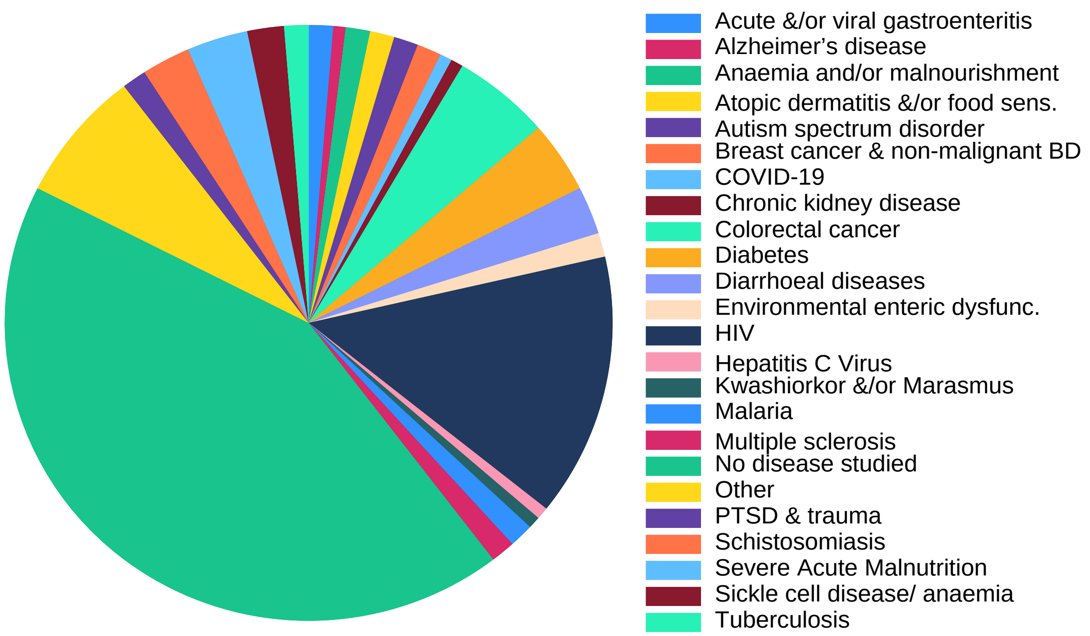


Other: A combination of different types of diseases, and those that appeared once and are also of not public health significance.

Of all the studies that were included in the current scoping review, only 11 of them studied diseases of public health significance (which were defined as top the causes of mortality in the African Region as listed by the (WHO), 2018), in relation to the gut microbiota. The diseases included Breast cancer, Colorectal cancer, Diabetes Mellitus, Diarrhoeal diseases, HIV, Malaria and Tuberculosis. The top 3 diseases that were mostly studied included HIV (14.3%), Colorectal cancer (5.2%) and Diabetes mellitus (3.9%), all of which formed part of the list of top causes of mortality in Africa. Human immunodeficiency virus was predominantly studied by South Africa (40.9%). Other countries that studied the infectious disease included Uganda (13.6%), Cameroon (9.1%), Mozambique (9.1%), Zimbabwe (9.1%), Ethiopia (4.5%), Ghana (4.5%), Kenya (4.5%) and Nigeria (4.5%). Colorectal cancer was mostly studied in Egypt (25%). Ethiopia (12.5%), Kenya (12.5%), Morocco (12.5%) Nigeria (12.5%) and Zimbabwe (12.5%) equally studied the condition as well, while the remaining 12.5% was accounted for by the “other” category. Fifty (50) percent of the Diabetes Mellitus studies came from Egypt. Other countries that also studied the disease were Nigeria (16.7%), Sudan (16.7%) and Tunisia (16.7%). The majority of the studies (42.9%) that were included in this review did not look at any disease in relation to the gut microbiota.

| Country of origin | Freq. | Percent | Cum. |
|---:|---:|---:|---:|
| Angola | 3 | 1.95 | 1.95 |
| Burkina Faso | 4 | 2.60 | 4.55 |
| Cameroon | 5 | 3.25 | 7.79 |
| Central African Republic | 1 | 0.65 | 8.44 |
| Côte d’Ivoire | 4 | 2.60 | 11.04 |
| Democratic Republic of the Congo | 1 | 0.65 | 11.69 |
| Egypt | 15 | 9.74 | 21.43 |
| Ethiopia | 2 | 1.30 | 22.73 |
| Gambia | 4 | 2.60 | 25.32 |
| Ghana | 6 | 3.90 | 29.22 |
| Guinea-Bissau | 1 | 0.65 | 29.87 |
| Kenya | 11 | 7.14 | 37.01 |
| Madagascar | 2 | 1.30 | 38.31 |
| Malawi | 16 | 10.39 | 48.70 |
| Mali | 3 | 1.95 | 50.65 |
| Morocco | 2 | 1.30 | 51.95 |
| Mozambique | 2 | 1.30 | 53.25 |
| Niger | 4 | 2.60 | 55.84 |
| Nigeria | 10 | 6.49 | 62.34 |
| Other | 6 | 3.90 | 66.23 |
| Senegal | 1 | 0.65 | 66.88 |
| Sierra Leone | 1 | 0.65 | 67.53 |
| South Africa | 25 | 16.23 | 83.77 |
| Sudan | 2 | 1.30 | 85.06 |
| Tanzania | 5 | 3.25 | 88.31 |
| Tunisia | 3 | 1.95 | 90.26 |
| Uganda | 7 | 4.55 | 94.81 |
| Zimbabwe | 8 | 5.19 | 100.00 |
| Total | 154 | 100.00 | |

.

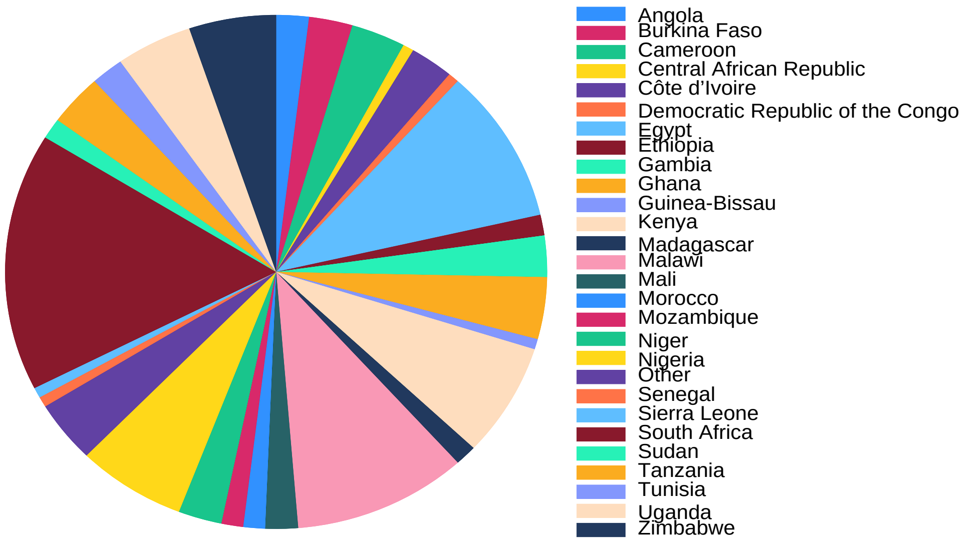


Other= A combination of different countries

The top 5 countries that contributed to gut microbiota research include South Africa (16.2%), Malawi (10.4%), Egypt (9.7%), Kenya (7.1%), Nigeria (6.5%).

| Study setting (urban/city or rural) | Freq. | Percent | Cum. |
|---|---|---|---|
| City | 3 | 1.95 | 1.95 |
| City and rural | 1 | 0.65 | 2.60 |
| Inner-city or suburban | 1 | 0.65 | 3.25 |
| Not indicated | 77 | 50.00 | 53.25 |
| Peri-urban | 5 | 3.25 | 56.49 |
| Rural | 43 | 27.92 | 84.42 |
| Rural and semi-urban | 1 | 0.65 | 85.06 |
| Rural, semi-rural and semi-urban | 1 | 0.65 | 85.71 |
| Rural, semi-urban and urban | 2 | 1.30 | 87.01 |
| Semi-rural | 1 | 0.65 | 87.66 |
| Semi-urban (Fulani)/nomadic (Jarawa) | 1 | 0.65 | 88.31 |
| Urban | 6 | 3.90 | 92.21 |
| Urban and rural | 6 | 3.90 | 96.10 |
| Village | 6 | 3.90 | 100.00 |
| Total | 154 | 100.00 | |

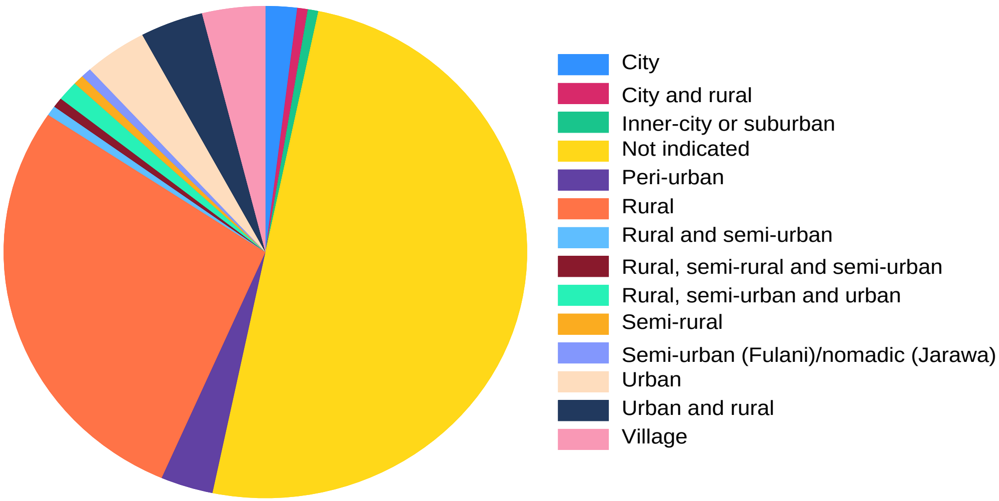


Half of the included studies (50%) did not indicate the type of study setting in which their research was conducted. When looking at those that indicated the study setting, majority of the them were conducted in the rural areas (27.9%).

| Methods/ technologies used to analyze the gut microbiota | Freq. | Percent | Cum. |
|---|---|---|---|
| 16S pyrosequencing and targeted real-.. | 2 | 1.30 | 1.30 |
| 16S rDNA sequencing | 5 | 3.25 | 4.55 |
| 16S rRNA/ metagenomic sequencing | 117 | 75.97 | 80.52 |
| Other | 15 | 9.74 | 90.26 |
| Quantitative PCR (qPCR)/ real time PCR | 15 | 9.74 | 100.00 |
| Total | 154 | 100.00 | |

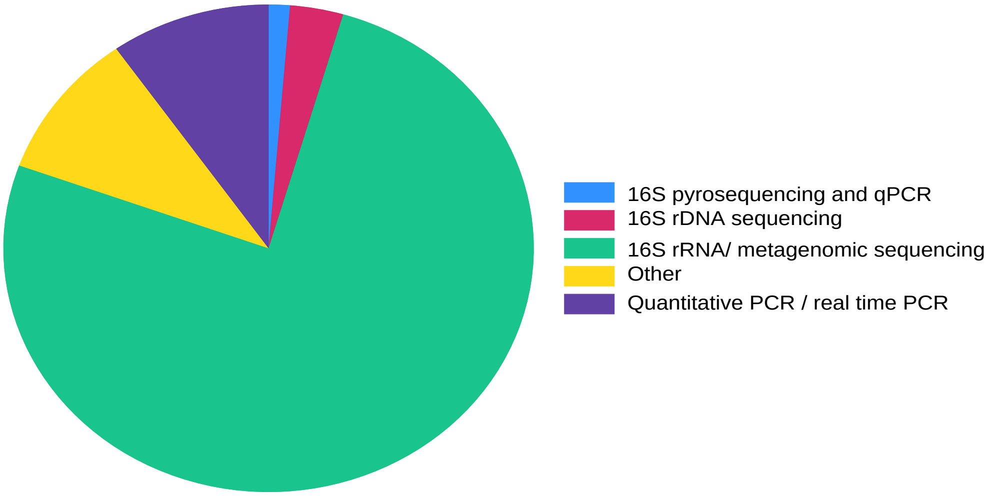

Other: A combination of more than one type of method used, and method types that appeared once.

The dominating method of choice for gut microbiota profiling was the 16S rRNA/ metagenomic sequencing (76.0%), which was followed by Quantitative PCR/ real time PCR (9.7%).

| Study design | Freq. | Percent | Cum. |
|---|---|---|---|
| Case-control study | 14 | 9.09 | 9.09 |
| Cohort study | 6 | 3.90 | 12.99 |
| Comparative study | 9 | 5.84 | 18.83 |
| Experimental/ clinical trial | 26 | 16.88 | 35.71 |
| Exploratory study | 1 | 0.65 | 36.36 |
| Follow-up study of a clinical trial | 1 | 0.65 | 37.01 |
| Longitudinal study | 8 | 5.19 | 42.21 |
| Observational/ cross-sectional study | 59 | 38.31 | 80.52 |
| Other | 6 | 3.90 | 84.42 |
| Pilot study | 7 | 4.55 | 88.96 |
| Pre-liminary study | 1 | 0.65 | 89.61 |
| Prospective study | 2 | 1.30 | 90.91 |
| Retrospective convenience sample design | 2 | 1.30 | 92.21 |
| Sub-study | 12 | 7.79 | 100.00 |
| Total | 154 | 100.00 | |

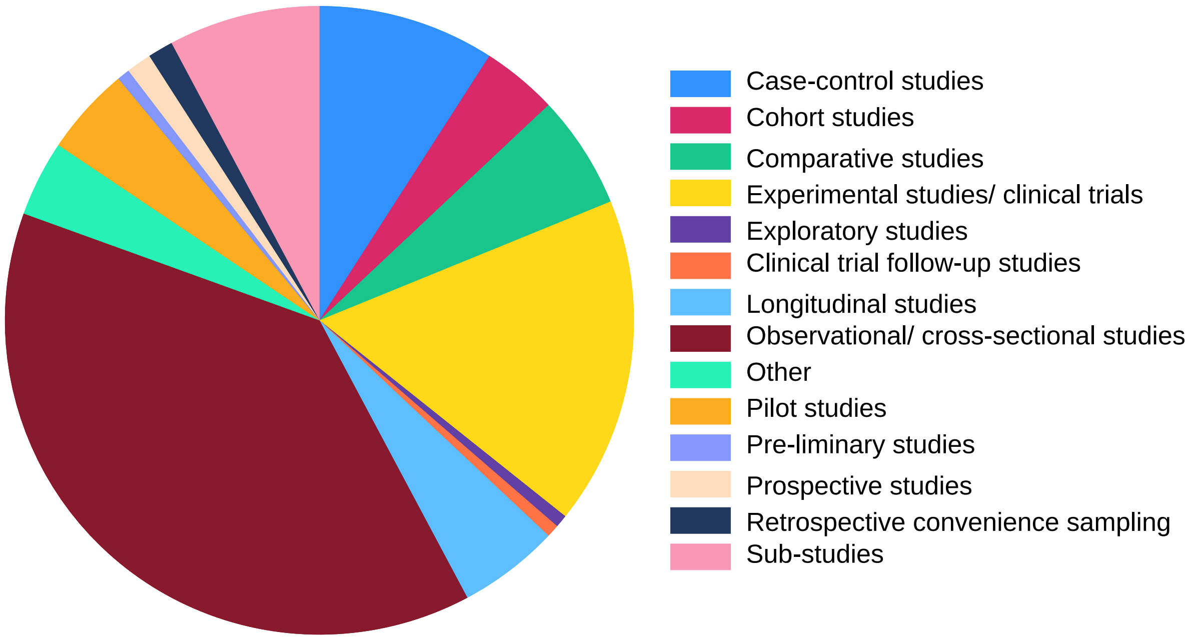


Other: A combination of more than one study design.

The predominant study designs used were Observational/ cross-sectional studies (38.3%), Experimental/ clinical trials (16.9%) and Case-control studies (9.1%).

| Type of sample used | Freq. | Percent | Cum. |
|---|---|---|---|
| Colonic mucosal samples/tissues | 5 | 3.25 | 3.25 |
| Rectal swabs/ sponges | 10 | 6.49 | 9.74 |
| Stool samples | 139 | 90.26 | 100.00 |
| Total | 154 | 100.00 | |

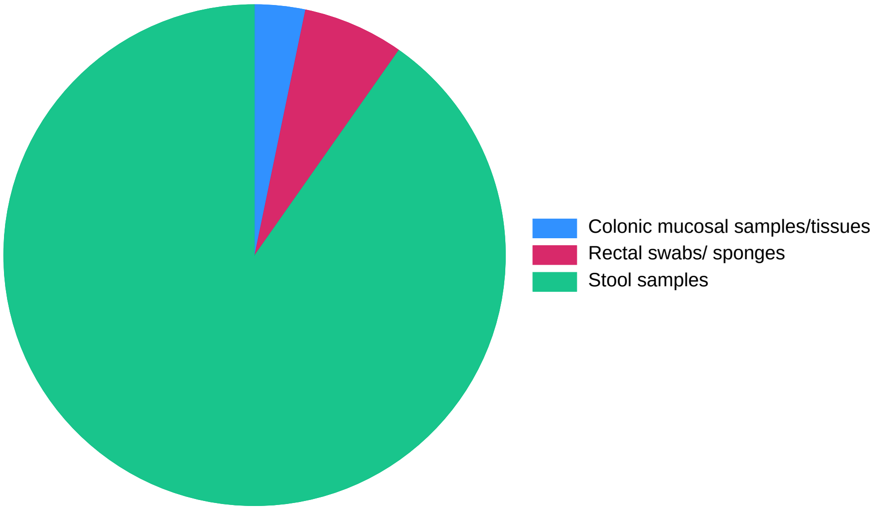


Majority of the included studies used stool samples for gut microbiota profiling (90.3%), while the rest made use of rectal swabs/sponges (6.5%) colonic mucosal samples/ tissues (3.3%).

| Population description | Freq. | Percent | Cum. |
|---|---|---|---|
| Adults | 43 | 27.92 | 27.92 |
| Children | 56 | 36.36 | 64.29 |
| Infants | 16 | 10.39 | 74.68 |
| Not indicated | 3 | 1.95 | 76.62 |
| Other | 35 | 22.73 | 99.35 |
| Toddlers | 1 | 0.65 | 100.00 |
| Total | 154 | 100.00 | |

| Race | Freq. | Percent | Cum. |
|---|---|---|---|
| Bambara | 1 | 0.65 | 0.65 |
| Black | 4 | 2.60 | 3.25 |
| Hadza | 1 | 0.65 | 3.90 |
| Mixed ancestry | 2 | 1.30 | 5.19 |
| Not indicated | 139 | 90.26 | 95.45 |
| Other | 7 | 4.55 | 100.00 |
| Total | 154 | 100.00 | |

| **Population group** | **Percentage (%)** |
|---|---|
| Adults | 27.9 |
| Children | 37.1 |
| Infants | 10.4 |
| Not indicated | 2.0 |
| Other | 22.7 |
| | |
| **Race/ ethnicity/ tribe** | **Percentage (%)** |
| Bambara | 0.7 |
| Black | 2.6 |
| Hadza | 0.7 |
| Mixed ancestry | 1.3 |
| Not indicated | 90.3 |
| Other | 4.6 |
| | |
| **Gender ratio (female/male) groups** | **N** |
| Ratios between 0.31- 0.99 | 35 |
| Ratios equal to 1 | 9 |
| Ratios between 1.05- 5.83 | 52 |
| Females only | 7 |
| Males only | 3 |
| Not indicated | 48 |
| | |
| **Age range:** | 0 months- 84 years |

Other in population group= A combination of different population groups.

Other in race= A combination of different races/ ethnic groups/ tribes.

The most studied population group were children (37.1%), which is followed by adults (27.9%) and then infants (10.4%). Moreover, majority of the studies (90.3%) did not mention the race/ ethnic groups/ tribes in which the research was conducted. Of the ethnicities that were mentioned, Blacks (2.6%) and mixed ancestry individuals (1.3%) were mostly studied as compared to the rest of the groups. Most of the studies were dominated by females as shown by the high number (n= 52) of ratios (female/male) between 1.05 and 5.83. However, 48 of the studies that were included in the current scoping review did not mention the number of males and females looked at. The age of the populations studied ranged from 0 moth to 84 years.

Sample sizes ranged from 3 to 1900. Of the studies that were included in this scoping review, 142 were scholarly articles, while 12 were published posters/abstracts.
