# Supplementary material for: A Scoping Review Evaluating the Current State of Gut Microbiota Research in Africa
Source: Microorganisms. 2023 Aug 20;11(8):2118. doi: 10.3390/microorganisms11082118 (PMC10458939; doi:10.3390/microorganisms11082118)
Supplement: Supplementary file 1 [file microorganisms-11-02118-s001.zip › Supplementary S3- search histories.pdf]

<u>SEARCH HISTORIES</u>

Scopus (Elseviers)

	**Interface - EBSCOhost Research Databases**<br>**Database - CINAHL Complete**
S3	S1 AND S2
S2	MH africa OR TI ((Nigeria OR Ethiopia OR Egypt OR Dr Congo OR Tanzania OR South Africa OR Kenya OR Uganda OR Sudan OR Algeria OR Morocco OR Angola OR Ghana OR Mozambique OR Madagascar OR Ivory Coast OR Cameroon OR Niger OR Mali OR Burkina Faso OR Malawi OR Zambia OR Chad OR Somalia OR Senegal OR Zimbabwe OR Guinea OR Rwanda OR Benin OR Burundi OR Tunisia OR South Sudan OR Togo OR Sierra Leone OR Libya OR Republic Of The Congo OR Central African Republic OR Liberia OR Mauritania OR Eritrea OR Gambia OR Botswana OR Namibia OR Gabon OR Lesotho OR Guinea Bissau OR Equatorial Guinea OR Mauritius OR Eswatini OR Djibouti OR Comoros OR Cape Verde OR Sao Tome And Principe OR Seychelles) OR AB ((Nigeria OR Ethiopia OR Egypt OR Dr Congo OR Tanzania OR South Africa OR Kenya OR Uganda OR Sudan OR Algeria OR Morocco OR Angola OR Ghana OR Mozambique OR Madagascar OR Ivory Coast OR Cameroon OR Niger OR Mali OR Burkina Faso OR Malawi OR Zambia OR Chad OR Somalia OR Senegal OR Zimbabwe OR Guinea OR Rwanda OR Benin OR Burundi OR Tunisia OR South Sudan OR Togo OR Sierra Leone OR Libya OR Republic Of The Congo OR Central African Republic OR Liberia OR Mauritania OR Eritrea OR Gambia OR Botswana OR Namibia OR Gabon OR Lesotho OR Guinea Bissau OR Equatorial Guinea OR Mauritius OR Eswatini OR Djibouti OR Comoros OR Cape Verde OR Sao Tome And Principe OR Seychelles)
S1	TX (gut microbiome OR gut microbiota OR gut bacteria OR gut micro flora OR enteric bacteria OR flora gut OR gastric microbiome * OR intestinal flora) OR MH gastrointestinal microbiome OR TX 16S rRNA

(TITLE-ABS-KEY (gut AND microbiome OR gut AND microbiota OR gut AND bacteria OR enteric AND bacteria OR flora AND gut OR gastric AND microbiome* OR intestinal AND flora OR gastrointestinal AND microbiome OR 16s AND rna) AND TITLE-ABS-KEY (africa* OR africa OR nigeria OR ethiopia OR egypt OR dr AND congo OR tanzania OR south AND africa OR kenya OR uganda OR sudan OR algeria OR morocco OR angola OR ghana OR mozambique OR madagascar OR ivory AND coast OR cameroon OR niger OR mali OR burkina AND faso OR malawi OR zambia OR chad OR somalia OR senegal OR zimbabwe OR guinea OR rwanda OR benin OR burundi OR tunisia OR south AND sudan OR togo OR sierra AND leone OR libya OR republic AND of AND the AND congo OR central AND african AND republic OR liberia OR mauritania OR eritrea OR gambia OR botswana OR namibia OR gabon OR lesotho OR guinea AND bissau OR equatorial AND guinea OR mauritius OR eswatini OR djibouti OR comoros OR cape AND verde OR (sao AND tome AND principe) OR seychelles))

Google scholar = gut microbiome africa*

WHO ICTRP = gut microbiome AND Africa*

Web of Science Core Collection

CPCI-S , SCI-EXPANDED

Search

#3 #1 AND #2

#2 africa* OR nigeria OR ethiopia OR egypt OR (dr AND congo) OR tanzania OR south AND africa OR kenya OR uganda OR sudan OR algeria OR morocco OR angola OR ghana OR mozambique OR madagascar OR ivory AND coast OR cameroon OR niger OR mali OR burkina AND faso OR malawi OR zambia OR chad OR somalia OR senegal OR zimbabwe OR guinea OR rwanda OR benin OR burundi OR tunisia OR south AND sudan OR togo OR sierra AND leone OR libya OR (republic AND congo) OR (central AND african AND republic) OR liberia OR mauritania OR eritrea OR gambia OR botswana OR namibia OR gabon OR lesotho OR guinea AND bissau OR equatorial AND guinea OR mauritius OR eswatini OR djibouti OR comoros OR cape AND verde OR (sao AND tome AND principe) OR seychelles (Topic)

#1 (gut microbiome OR gut microbiota OR gut bacteria OR gut micro flora OR enteric bacteria OR flora gut OR gastric microbiome * OR intestinal flora (Topic) or 16S rRNA (Topic)

Pubmed (Medline)

1 (((africa[MeSH Terms]) OR ((Nigeria[Title/Abstract] OR Ethiopia[Title/Abstract] OR Egypt[Title/Abstract] OR Dr Congo[Title/Abstract] OR Tanzania[Title/Abstract] OR South Africa[Title/Abstract] OR Kenya[Title/Abstract] OR Uganda[Title/Abstract] OR Sudan[Title/Abstract] OR Algeria[Title/Abstract] OR Morocco[Title/Abstract] OR Angola[Title/Abstract] OR Ghana[Title/Abstract] OR Mozambique[Title/Abstract] OR Madagascar[Title/Abstract] OR Ivory Coast[Title/Abstract] OR Cameroon[Title/Abstract] OR Niger[Title/Abstract] OR Mali[Title/Abstract] OR Burkina Faso[Title/Abstract] OR Malawi[Title/Abstract] OR Zambia[Title/Abstract] OR Chad[Title/Abstract] OR Somalia[Title/Abstract] OR Senegal[Title/Abstract] OR Zimbabwe[Title/Abstract] OR Guinea[Title/Abstract] OR Rwanda[Title/Abstract] OR Benin[Title/Abstract] OR Burundi[Title/Abstract] OR Tunisia[Title/Abstract] OR South Sudan[Title/Abstract] OR Togo[Title/Abstract] OR Sierra Leone[Title/Abstract] OR Libya[Title/Abstract] OR Republic Of The Congo[Title/Abstract] OR Central African Republic[Title/Abstract] OR Liberia[Title/Abstract] OR Mauritania[Title/Abstract] OR Eritrea[Title/Abstract] OR Gambia[Title/Abstract] OR Botswana[Title/Abstract] OR Namibia[Title/Abstract] OR Gabon[Title/Abstract] OR Lesotho[Title/Abstract] OR Guinea Bissau[Title/Abstract] OR Equatorial Guinea[Title/Abstract] OR Mauritius[Title/Abstract] OR Eswatini[Title/Abstract] OR Djibouti[Title/Abstract] OR Comoros[Title/Abstract] OR Cape Verde[Title/Abstract] OR Sao Tome[Title/Abstract] AND Principe[Title/Abstract] OR Seychelles[Title/Abstract])) OR (africa*[Title/Abstract])) AND ((((gut microbiome[Title/Abstract] OR gut microbiota[Title/Abstract] OR gut bacteria[Title/Abstract] OR gut microflora[Title/Abstract] OR enteric bacteria[Title/Abstract] OR flora gut[Title/Abstract] OR gastric microbiome *[Title/Abstract] OR intestinal flora[Title/Abstract]) OR ("Gastrointestinal Microbiome"[Mesh])) OR (16S rRNA[Title/Abstract])) OR ("RNA, Ribosomal, 16S"[Mesh]))

Embase 1947-Present, updated daily

1	(gut microbiome or gut microbiota or gut bacteria or gut micro flora or enteric bacteria or flora gut or gastric microbiome* or intestinal flora or gastrointestinal microbiome or 16S rRNA).ti,ab.
2	intestine flora/
3	africa.mp. or "Africa south of the Sahara"/ or South Africa/ or Africa/ or North Africa/ or Central Africa/
4	(Nigeria or Ethiopia or Egypt or Congo or Tanzania or South Africa or Kenya or Uganda or Sudan or Algeria or Morocco or Angola or Ghana).mp.
5	(Mozambique or Madagascar or Ivory Coast or Cameroon or Niger or Mali).mp.
6	(Burkina Faso or Malawi or Zambia or Chad or Somalia or Senegal or Zimbabwe or Guinea or Rwanda or Benin or Burundi).mp.
7	(Tunisia or South Sudan or Togo or Sierra Leone or Libya).mp.
8	(Central African Republic or Liberia or Mauritania or Eritrea or Gambia or Botswana or Namibia or Gabon or Lesotho).mp.
9	(Guinea Bissau or Equatorial Guinea or Mauritius or Eswatini or Djibouti or Comoros or Cape Verde).mp.
10	((Sao Tome and Principe) or Seychelles).mp.
11	3 or 4 or 5 or 6 or 7 or 8 or 9 or 10
12	1 or 2
13	limit 12 to human
14	11 and 13
